# Inhibitory effects of corylin derived from aerial part of *Pueraria lobata* on melanin synthesis and potential applications in skin whitening and photoaging management

**DOI:** 10.1007/s13659-025-00509-8

**Published:** 2025-04-27

**Authors:** BoYoon Chang, SungYeon Kim

**Affiliations:** Institute of Pharmaceutical Research and Development, College of Pharmacy, Wonkwang University, Iksan, Jeonbuk 54538 Republic of Korea

**Keywords:** Corylin, 3D skin model, Melanogenesis, Aerial part of *Pueraria lobata*

## Abstract

**Purpose:**

This study aimed to investigate the potential of corylin, a bioactive compound isolated from the aerial part of *Pueraria lobata*, as a novel skin-whitening agent. Specifically, the research sought to evaluate its effects on melanin synthesis, understand its underlying mechanisms, and validate its efficacy in mitigating hyperpigmentation.

**Methods:**

Bioactive compound was isolated from *Pueraria lobata* through a systematic fractionation process involving activated carbon pigment removal, sequential solvent extraction, and resin-based chromatography. It was shown to inhibit melanin synthesis by targeting tyrosinase activation and modulating key signaling pathways. Its efficacy in reducing melanin production was validated through cellular assays and a UVB-stimulated 3D human skin model, highlighting its potential as a skin-whitening agent.

**Results:**

Through fractionation, the bioactive compound was identified as corylin, which reduced melanin content and tyrosinase activity without cytotoxicity, modulated signaling pathways to downregulate MITF and melanogenic enzymes, and inhibited α-glucosidase, disrupted glycosylation. In a UVB-stimulated 3D skin model, it effectively decreased melanin production, confirming its potential to mitigate hyperpigmentation.

**Conclusion:**

Corylin is a promising candidate for skin-whitening applications, effectively mitigating hyperpigmentation by targeting multiple stages of melanin synthesis, including enzymatic activity and regulatory pathways. Further clinical studies are needed to confirm its safety and therapeutic potential for dermatological use.

**Graphical Abstract:**

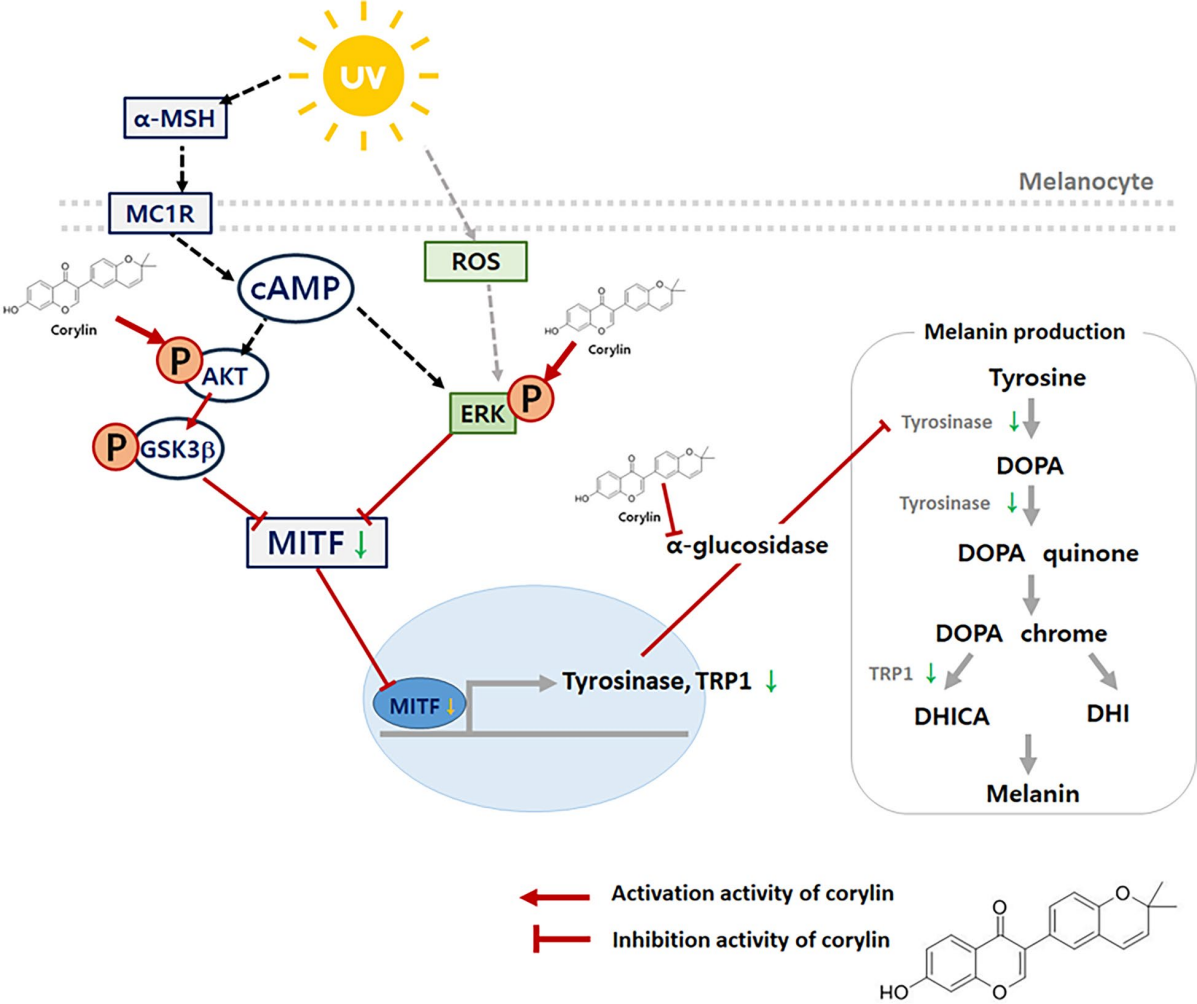

## Introduction

*Pueraria* species is a medicinal plant traditionally recognized for its health promoting and disease preventing properties. Various bioactive compounds, such as puerarin, daidzein, glycitein, genistein, formononetin, and biochanin A, have been identified in its roots and flower through extensive research [1–4]. However, the aerial parts of *Pueraria* species remain underexplored, with their pharmacological potential yet to be fully investigated.

The vines of *Pueraria* species are often regarded as waste material due to their rapid growth and substantial biomass, which can cause significant damage to forests and landscapes. In certain regions, these species are classified as invasive, posing a serious threat to ecosystems [5]. Their management requires considerable financial resources and labor, highlighting the challenges associated with controlling their spread and mitigating their ecological impact.

Our research team has pioneered the systematic investigation of the biological activities of the aerial parts of *Pueraria* species, focusing on their untapped pharmacological potential. Preliminary studies revealed that the aerial parts of *P. lobata* exhibit diverse biological activities, including antioxidant, anti-inflammatory [5], hepatoprotective [6], and skin-whitening effects. Among these, the skin-whitening activity has garnered particular attention. In previous our studies, the ethyl acetate fraction on aerial parts of *P. thunbergiana* demonstrated an inhibitory effect on melanin production in B16F10 melanoma cells and UV-irradiated animals [7]. The ethyl acetate (EtOAc) fraction on aerial parts of *P. lobata* (APPL) demonstrated an inhibitory effect on melanin production in B16F10 melanoma cells and its cosmetic formulation was validated through clinical trials to significantly improve hyperpigmentation while ensuring dermatological safety [8].

However, the specific bioactive compounds responsible for this activity were not identified. Melanin synthesis and skin pigmentation are primarily regulated by key melanogenic enzymes, including tyrosinase, tyrosinase-related protein-1 (TRP-1), and tyrosinase-related protein-2 (TRP-2), which are localized within melanosomes. These enzymes are transcriptionally regulated by the microphthalmia-associated transcription factor (MITF), a master regulator of melanogenesis. MITF controls the expression of tyrosinase, TRP-1, and TRP-2 in response to various external stimuli, including UV radiation and hormonal signals. Activation of MITF occurs primarily through the cAMP/PKA/CREB pathway and the Wnt/β-catenin pathway, which play crucial roles in melanocyte function and pigment production [9]. Understanding the mechanisms that inhibit melanin production and identifying bioactive compounds involved are critical for advancing dermatological research and developing cosmetic applications.

The APPL was fractionated to isolate and identify major bioactive compounds responsible for skin-whitening activity. Furthermore, we investigated the mechanisms underlying their anti-melanogenic effects. The isolated bioactive compound (Corylin) was further investigated individually to elucidate their whitening mechanisms. Additionally, the whitening efficacy of these compounds was assessed using the artificial human pigmented skin model, Neoderm-ME, as a reliable alternative to in vivo experiments.

## Results

### Effects of APPL fractions on anti-melanogenesis in B16F10 cells

#### Isolation and identification of corylin

**Fig. 1:**
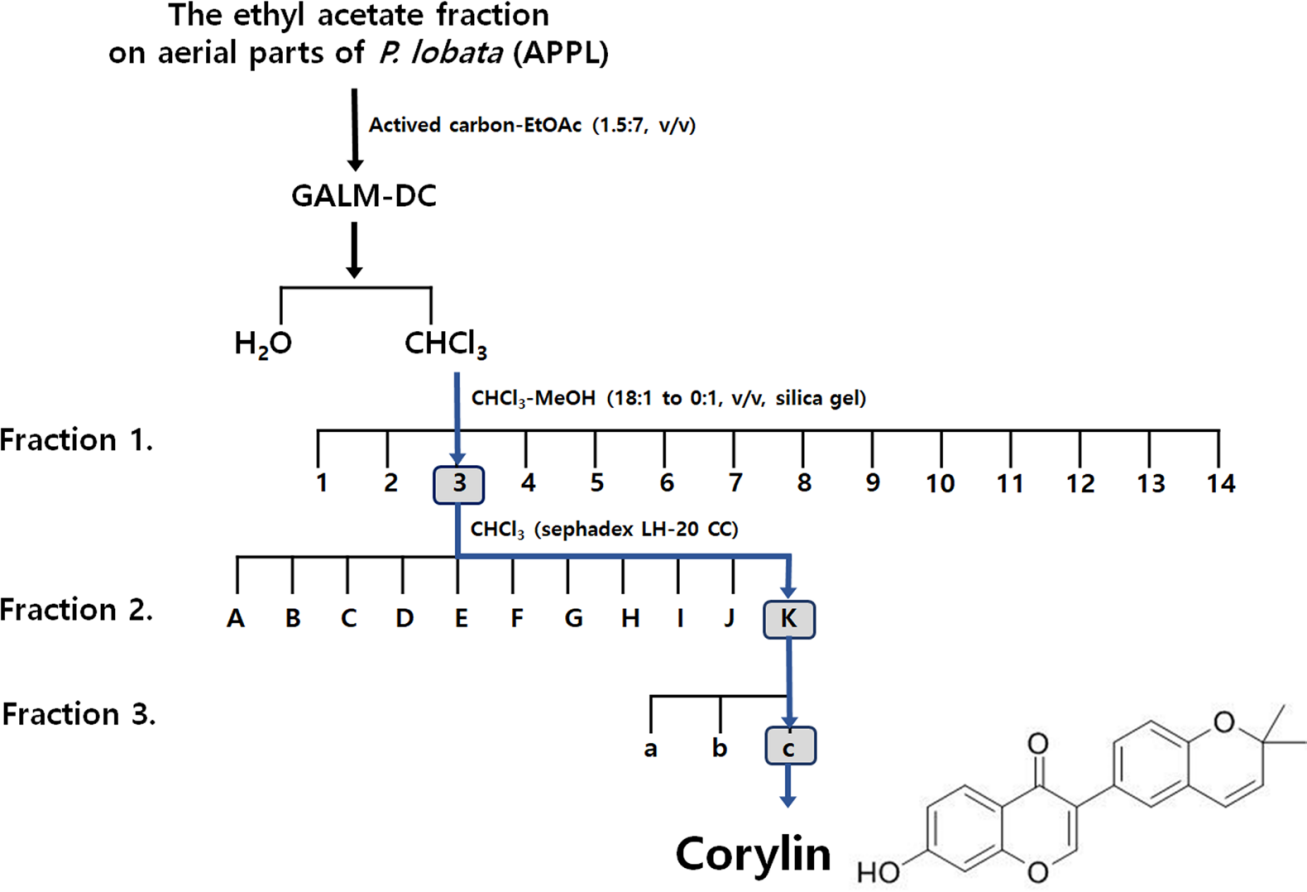
Fractionation diagram of the aerial parts of *Pueraria lobata* (APPL) and the chemical structure of corylin isolated from APPL

The aerial part of *Pueraria lobata* was fractionated with ethyl acetate (EtOAc) to obtain the extract (APPL). APPL underwent a decolorization process and three stages of fractionation, ultimately leading to the isolation of corylin as the active compound (Fig. 1).

#### Effect of APPL fractions on Cytotoxicity

After evaluating the cytotoxicity of fractions, melanin synthesis was induced using α-MSH at non-toxic concentrations, and the inhibitory effects of each fraction on melanin production were assessed.

**Fig. 2:**
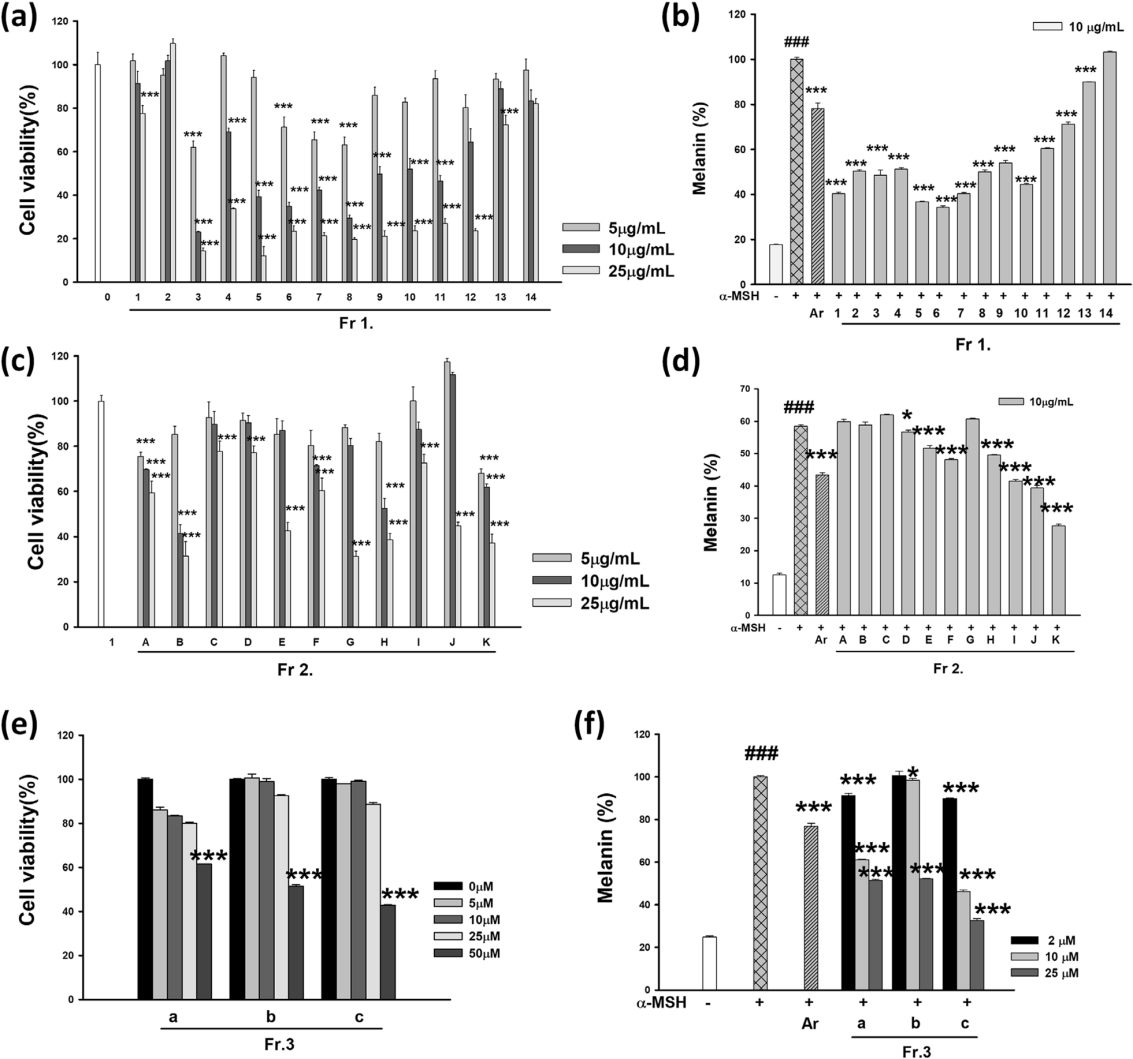
Effects of fractions from the aerial parts of *Pueraria lobata* (APPL) on anti-melanogenesis in B16F10 cells. (**a**, **b**) show the cell viability and melanin content of 14 fractions from the first fractionation of APPL using Sephadex LH-20 column chromatography with CHCl₃. (**c**, **d**) represent the results for 11 fractions further separated from Fr. 3 using silica gel chromatography with a CHCl₃-MeOH gradient (18:1 to 0:1, v/v). (e,f) display the data for 3 fractions derived from Fr. 3.K through silica gel column chromatography with hexane–EtOAc (4:1, v/v). Data are presented as mean ± SD. ^###^*p* < 0.001 compared with the compared with untreated group. *p < 0.05 and *** p < 0.001 compared with the compared with cells stimulated with α-MSH alone

The cytotoxicity of Fr. 1 fractions (1–14) was evaluated at concentrations of 5, 10 and 25 μg/mL. A dose-dependent decrease in cell viability was observed, with certain fractions (Fr. 3, 6, 8, and 12) exhibiting significant cytotoxicity at concentrations of 25 μg/mL or higher, reducing cell viability below 50% (Fig. 2a). Based on these findings, the melanin inhibition assay was conducted at a non-toxic concentration of 10 μg/mL.

The cytotoxicity of Fr. 2 subfractions (A–K) was assessed at 5, 10, and 25 μg/mL. While the control group maintained 100% cell viability, certain subfractions showed a dose-dependent decrease in cell viability (Fig. 2c). In particular, B, C, E, G, H, and K exhibited significant cytotoxicity at concentrations ≥ 25 μg/mL, reducing cell viability below 50%. Therefore, the melanin inhibition assay was performed at a non-toxic concentration of 10 μg/mL.

The cytotoxicity of Fr. 3 subfractions (a, b, and c) was analyzed at 5, 10, 25, and 50 μM. A concentration-dependent reduction in cell viability was observed across all subfractions (Fig. 2e). Fr. 3.c exhibited significant cytotoxicity at ≥ 25 μM, with cell viability decreasing below 50%. Fr. 3.a and Fr. 3.b also showed reduced viability at 50 μM; however, at lower concentrations (5–10 μM), cytotoxicity was minimal. Based on these results, melanin synthesis inhibition assays were conducted at non-toxic concentrations of 2, 10, and 25 μM.

The melanin synthesis inhibition assay was conducted at non-toxic concentrations determined from the cytotoxicity evaluation. The α-MSH-treated control group exhibited a significant increase in melanin production (^###^*p* < 0.001), which was used as a baseline for evaluating the melanin inhibitory effects of the fractions.

#### Effect of APPL fractions on anti-melanogenesis 

In the Fr. 1, fractions 1–10 demonstrated significant melanin inhibition, with suppression rates ranging from 40 to 60% (, p < 0.001) at 10 μg/mL (Fig. 2b). Notably, fractions 3, 5, 7, and 8 exhibited stronger inhibitory effects than the positive control, arbutin (100 μM), which showed an inhibition rate of 21.9 ± 2.5%. In contrast, fractions 11–14 did not exhibit significant melanin suppression, and some even showed melanin levels comparable to or higher than those of the α-MSH-treated control.

In the Fr. 2, melanin inhibition was evaluated at 10 μg/mL, the concentration determined to be non-toxic (Fig. 2d). Subfractions F, G, H, I, J, and K significantly inhibited melanin synthesis, with subfraction K exhibiting the most potent effect. Conversely, subfractions A–E showed little to no melanin suppression, with B, C, and D displaying melanin levels comparable to the α-MSH-treated control.

In the Fr. 3 series, melanin inhibition was assessed at 2, 10, and 25 μM, based on their cytotoxicity profiles (Fig. 2f). Among the subfractions, Fr. 3.c exhibited the strongest, dose-dependent melanin suppression (***, p < 0.001). While Fr. 3.a and Fr. 3.b also showed inhibitory effects, their potency was relatively lower than that of Fr. 3.c. At 25 μM, Fr. 3.c displayed the most significant reduction in melanin synthesis, suggesting its potential as a promising candidate for further studies on skin whitening applications.

### Effects of corylin on anti-melanogenesis in B16F10 cells

**Fig. 3:**
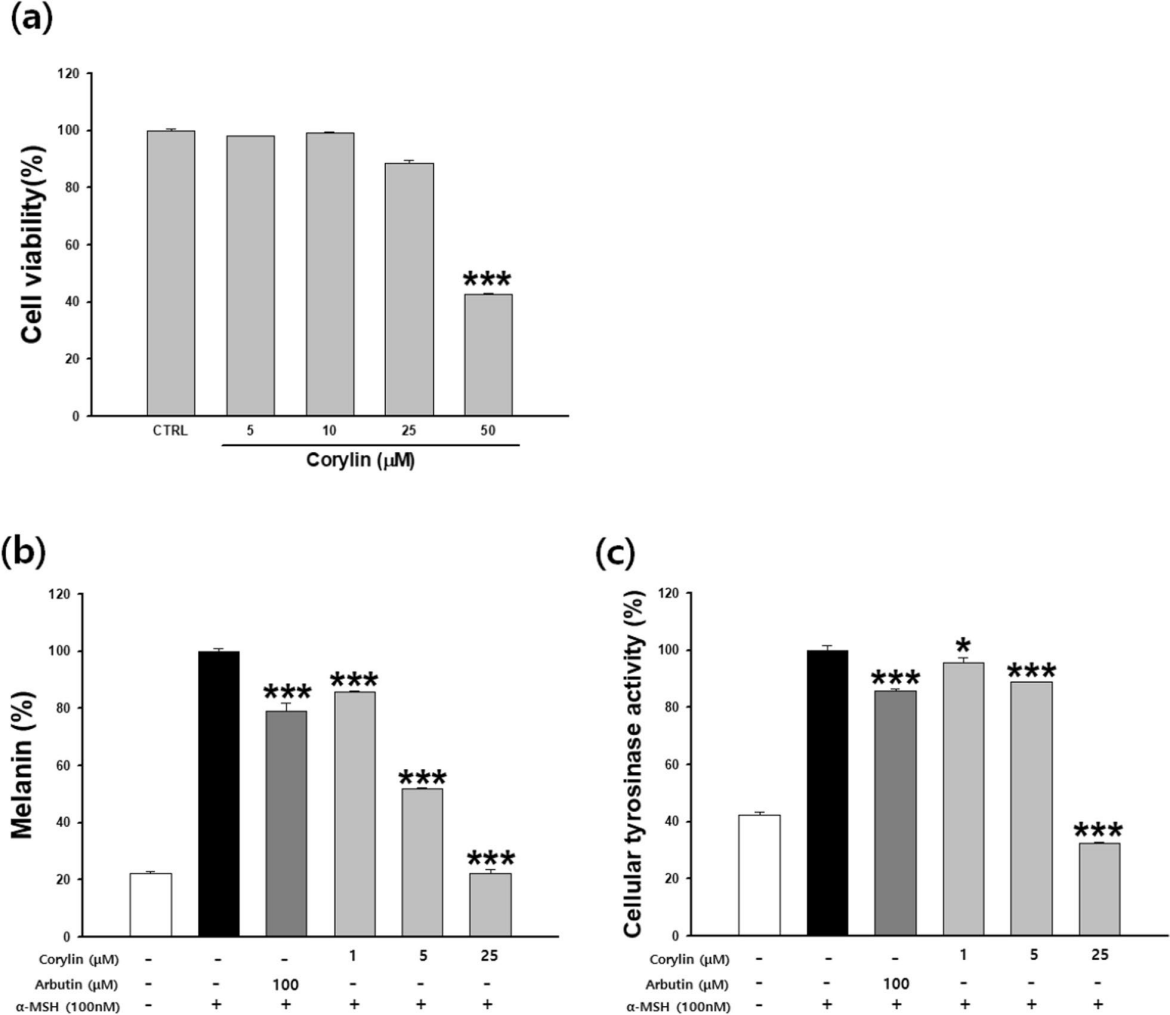
Effects of corylin on anti-melanogenesis in B16F10 cells. Cells were cultured with corylin (1–50 μM) for 48 h. (**a**) Cytotoxicity, (**b**) melanin contents, and (**c**) cellular tyrosinase activity were measured. Data are presented as mean ± SD. *p < 0.05 and *** p < 0.001 compared with the control (**a**) or compared with cells stimulated with α-MSH alone (**b**, **c**)

The B16F10 melanoma cells were treated with corylin at concentrations of 0–50 µM for 48 h. As corylin showed no cytotoxicity at 25 μM in B16F10 cells (Fig. 3a), corylin was thus treated with 1–25 μM. Increased melanin production was confirmed by α-MSH treatment. 100 μM arbutin was used as a positive control and 21% of melanin production was suppressed. Corylin decreased the melanin production increased by α-MSH in a concentration-dependent manner. 5 and 25 μM corylin inhibited melanin production by 48.2 and 77.9%, respectively (Fig. 3b). The cellular tyrosinase inhibition rates of corylin were 11.2% and 67.7% at 5 and 25 μM, respectively, compared to the α-MSH treated group. The arbutin group also inhibited the cellular tyrosinase rate by 14.3% (Fig. 3c). Corylin showed stronger anti-melanin activity than the positive control.

### Effects of corylin on melanogenesis signaling pathways in B16F10 cells

**Fig. 4:**
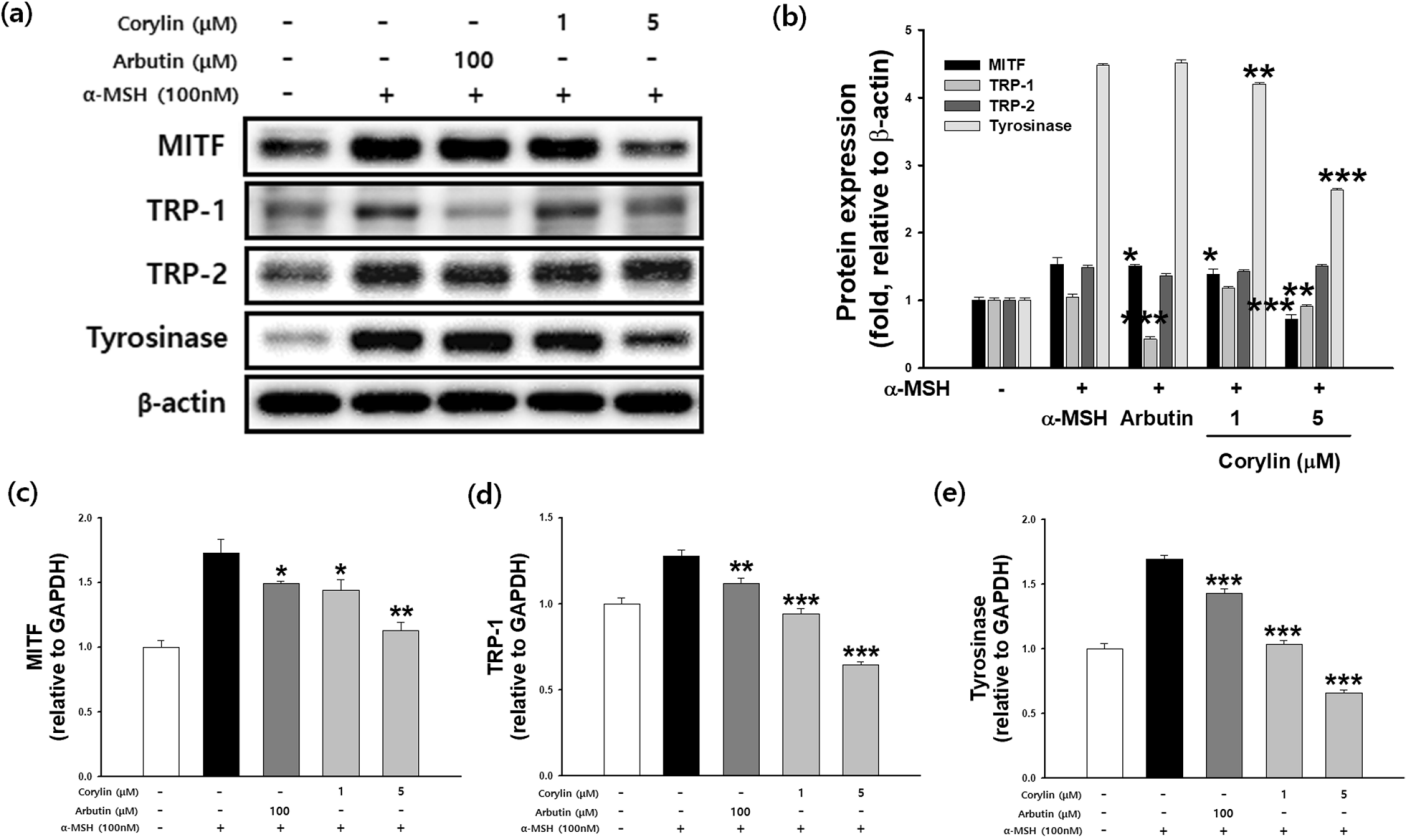
Effects of corylin on the expression of melanogenesis-related mRNA and proteins. B16F10 cells were treated with α-MSH and corylin at the indicated concentration. (**a**, **b**) MITF, TRP1, TRP2 and tyrosinase protein expression levels, and (**c**) MITF mRNA levels, (**d**) TRP1 mRNA levels, (**e**) tyrosinase mRNA levels. *p < 0.05, **p < 0.01, and ***p < 0.001 compared with cells stimulated with α -MSH alone

To investigate whether the inhibitory effect of corylin is related to the melanogenesis signaling pathway, cells were treated with corylin following stimulation with α-MSH. As shown in Fig. 4a, b, the protein levels of MITF, TRP1, and tyrosinase were reduced in a concentration-dependent manner by corylin compared to the α-MSH group. However, the expression level of TRP2 remained unaffected by corylin treatment. These results indicate that corylin inhibits melanogenesis by decreasing MITF, TRP1, and tyrosinase expression, while having no significant effect on TRP2. The mRNA and protein expression levels of MITF, tyrosinase, and TRP-1 were examined by real time-PCR and western blotting, respectively. The expression of MITF, TRP-1, and tyrosinase increased following treatment with 100 nM α-MSH. The inhibition rate of MITF expression was 16.7 and 34.7% at 1 and 5 μM corylin, respectively, compared to α-MSH treatment group.

At 1 and 5 μM, TRP-1 expression was inhibited by 26.1 and 49.6%, respectively. Tyrosinase expression was inhibited by 38.8 and 61.2% at 1 and 5 µM, respectively. The transcription of MITF, TRP1, and tyrosinase was significantly attenuated in a dose-dependent manner (Fig. 4c–e).

### Effects of corylin on signal transduction pathways in B16F10 cells

**Fig. 5:**
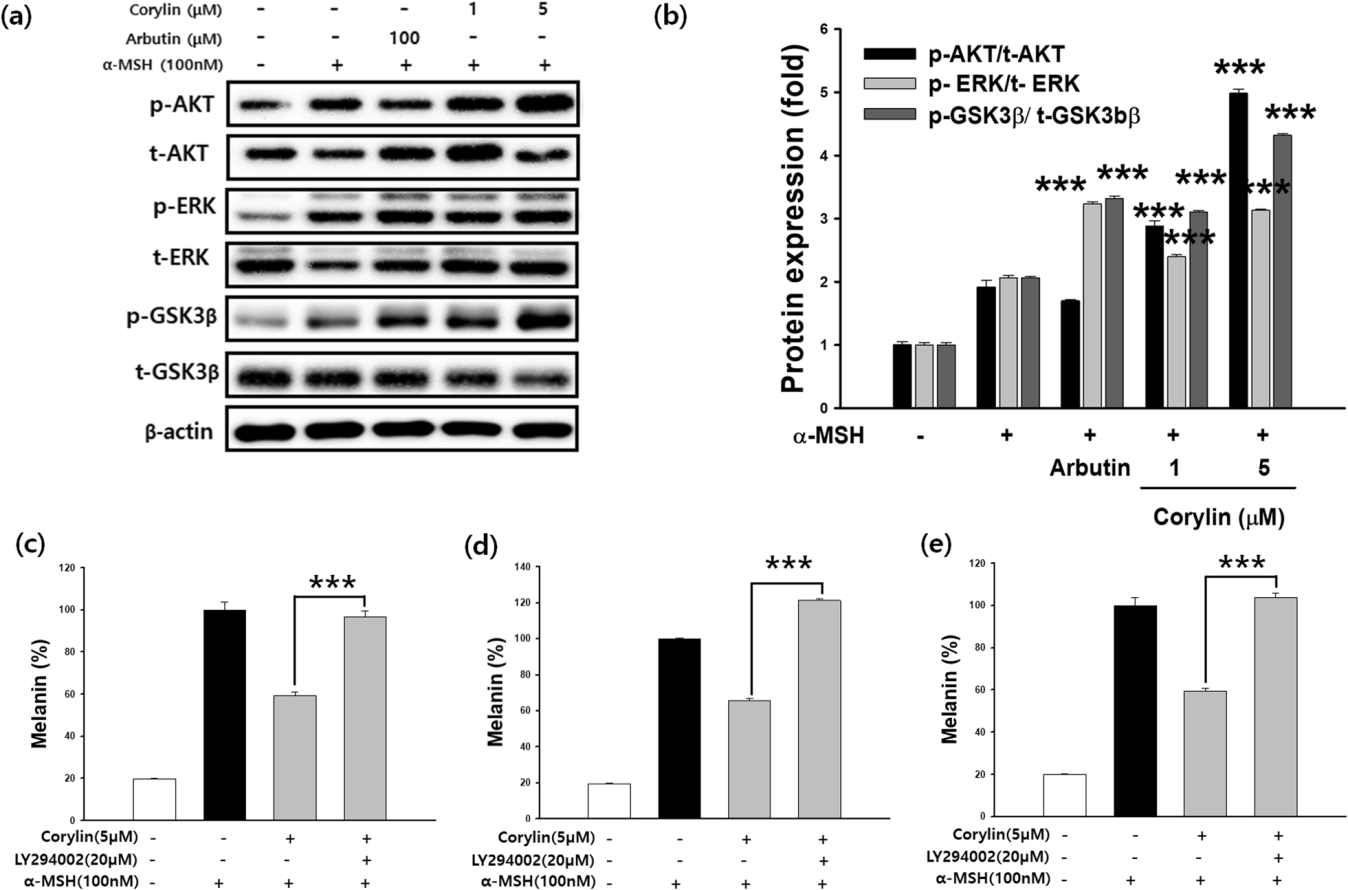
Effects of Corylin on PI3K/Akt/GSK-3β and MEK/ERK Signaling Pathways in B16F10 Cells. (**a**, **b**) B16F10 cells were treated with α-MSH (100 nM) and corylin at the indicated concentration to investigate its effect on Akt, GSK-3β, and ERK dependent signaling pathways. Additionally, cells were co-treated with corylin (5 µM) and selective inhibitors targeting specific pathways: (**c**) PI3K/Akt (LY294002), (**d**) GSK-3β (SB216763), and (**e**) MEK/ERK (PD98059) at specified concentrations (20 or 50 µM). Corylin significantly inhibited melanin production in α-MSH-stimulated B16F10 cells by regulating the MEK/ERK and PI3K/Akt signaling pathways. Co-treatment with selective inhibitors reversed the melanin-inhibitory effects of corylin, confirming the involvement of these pathways in corylin-mediated melanogenesis regulation. ***p < 0.001 compared with α-MSH and corylin co-treated cells

The PI3K/Akt/GSK-3β and MEK/ERK signaling pathways are crucial regulators of melanogenesis. To determine whether corylin influences these pathways, western blot analysis was conducted. Following corylin treatment, an increase in the phosphorylation of ERK, Akt, and GSK-3β was observed, indicating activation of these signaling pathways (Fig. 5a, b). To further investigate the role of these pathways in corylin-mediated melanogenesis inhibition, B16F10 cells were co-treated with corylin and specific inhibitors targeting PI3K (LY294002), GSK-3β (SB216763), or ERK (PD98059). The melanin content was subsequently measured. As shown in Fig. 5c–e, corylin treatment significantly attenuated α-MSH-induced melanogenesis. However, co-treatment with the specific inhibitors reversed the melanin-reducing effect of corylin, confirming that the PI3K/Akt/GSK-3β and MEK/ERK pathways are involved in the inhibitory effects of corylin on melanin synthesis.

### Inhibitory effect of corylin on glucosidase activity

**Fig. 6:**
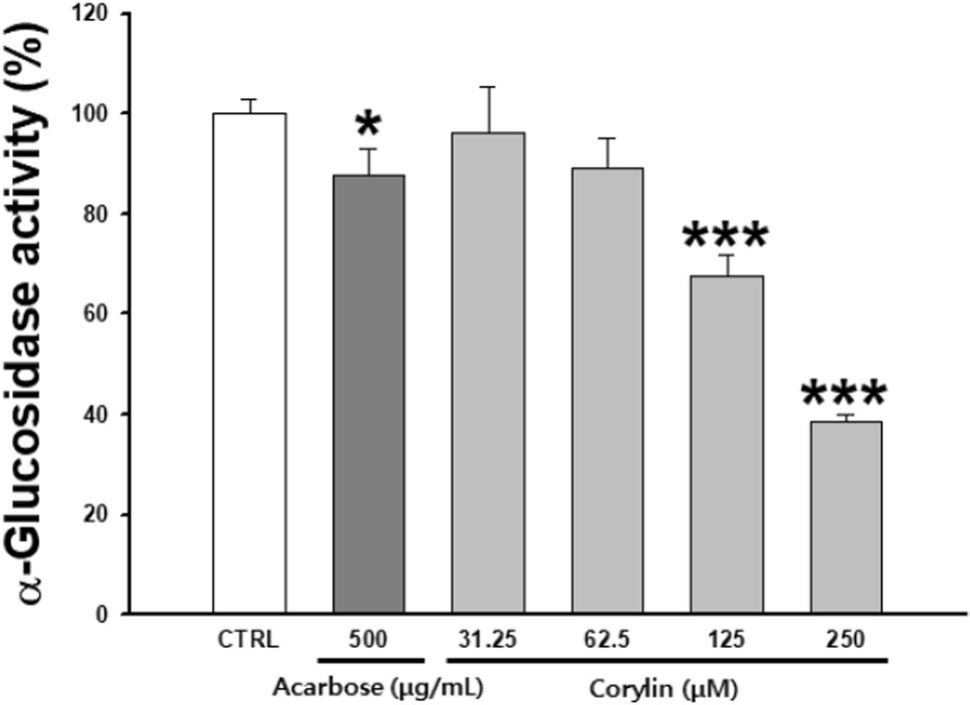
Inhibitory effects on α-glucosidase activity. Each percentage value representing α-glucosidase activity is reported relative to that of the control. Data are presented as mean ± SD. *p < 0.05 and *** p < 0.001 compared with the control

α-Glucosidase is one of many enzymes involved in the glycosylation process. When α-glucosidase is inhibited, the structure of tyrosinase is transformed and it migrates to melanosomes in an inactive form, resulting in inhibition of melanogenesis. Inhibition of glycosylation inhibits proper tyrosinase maturation and subsequent enzymatic activity. To confirm the inhibition of the α-glucosidase activity of corylin, it was performed in a cell-free system. Corylin showed 32.3 and 61.4% α-glucosidase inhibition rates at 125 and 250 μM, respectively. In the positive control, 500 μg/mL acarbose, α-glucosidase inhibition was 12.1%. Corylin showed a higher effect than acarbose (Fig. 6).

### Effect of corylin on melanin production in a reconstructed 3D skin model

**Fig. 7:**
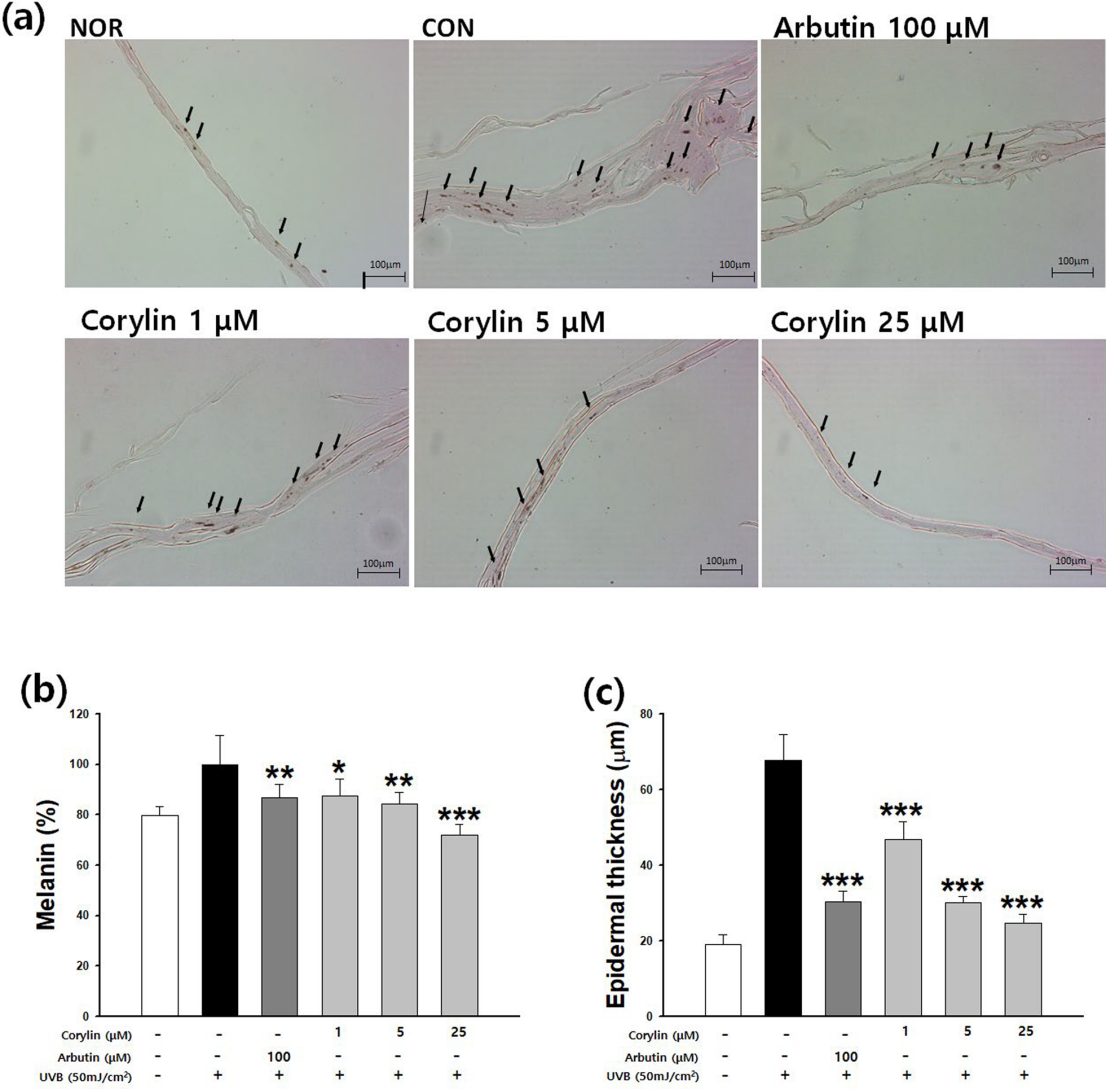
The inhibitory effect of corylin on melanin production in a reconstructed 3D skin model. (**a**) Fontana-Mason (F-M) staining of tissue sections, (**b**) Melanin content in a 3D pigmented human skin model, (**c**) Measurement of epidermal thickness. *p < 0.05, ** p < 0.01, and *** p < 0.001 compared with the UVB group

F-M staining was performed to observe the change in melanogenesis by corylin in a reconstructed human 3D skin model (Fig. 7a). Melanin production was increased in UVB-stimulated 3D human skin, and corylin significantly reduced melanin content. The UVB-irradiated group presented increased epidermal thickness compared with the normal group, whereas the corylin and arbutin treated groups presented a significant decrease in skin thickness (Fig. 7b). As a result of quantifying total melanin production, the amounts of melanin were dose-dependently reduced following treatment with corylin (Fig. 7c). The rate of inhibition with corylin was 15.7 and 27.9% at 5 and 25 µM, respectively, compared with the UVB-treated group. Inhibition of melanogenesis was equivalent to or greater than that of arbutin 100 µM (13.3%). These results suggest that corylin has an anti-melanogenic effect on human skin.

## Discussion

The fractionation of plant extracts is a critical process for isolating bioactive compounds and facilitating their study. Before fractionation, the ethyl acetate (EtOAc) fraction on aerial parts of *P. lobata* (APPL) exhibited a deep brown color [8, 10]. To facilitate the identification of bioactive compounds, the activated carbon method [11] was employed to effectively remove the intense pigmentation. By leveraging the high porosity and adsorption efficiency of activated carbon, pigment components were selectively removed while preserving the integrity of the active compounds.

This procedure employs a systematic approach, utilizing solvents and resins selected based on the polarity and chemical properties of the target compounds. Sequential extraction is performed with solvents of varying polarity, such as hexane, chloroform, ethyl acetate, and methanol, to effectively separate compounds [12, 13]. Subsequently, resin-based chromatography further refines the fractions. Resins like Sephadex LH-20 [14], silica gel, C18 reversed-phase resin, and Amberlite are chosen according to the chemical nature of the compounds being targeted [15].

Gradient elution with solvents ensures efficient separation, particularly for compounds with overlapping polarities. The collected fractions are analyzed using techniques such as TLC or HPLC, and concentrated fractions are prepared for further applications in pharmacological and cosmetic research [16].

Nuclear Magnetic Resonance (NMR) spectroscopy plays a crucial role in determining the structure of isolated active compounds. This technique provides comprehensive information about molecular characteristics, such as purity, functional group arrangement, and stereochemical configuration. By examining nuclei like hydrogen (^1^H) and carbon (^13^C), NMR reveals the chemical surroundings and bonding patterns within the molecule. Advanced methods, including 2D-NMR techniques such as COSY, HSQC, and HMBC, enable the analysis of intricate structures and molecular interactions, offering a detailed understanding of the compound's chemical attributes and biological functions [17].

Due to increasing societal interest, there has been a growing effort to identify active compounds with skin-whitening and anti-aging properties [18]. In this study, the skin-whitening potential of fractions isolated from APPL was evaluated based on the reduction of intracellular melanin content in B16F10 cells, without inducing cytotoxicity. The B16F10 cell line is a widely accepted model for studying melanin production and pigment reduction, as it requires stimulation by hormones capable of inducing melanin synthesis [19]. α-MSH, a key regulator of melanin synthesis, enhances tyrosinase activity and promotes the expression of TRP-1 and TRP-2, enzymes involved in eumelanin synthesis. Tyrosinase is a critical enzyme in melanocytes, playing an essential role in the production of both eumelanin and pheomelanin [7, 8]. In this study, significant reductions in melanin content were observed in fractions Fr. 3, 11, and 3, highlighting their potential as skin-whitening agents.

The final compound selected through fractionation of APPL, corylin, has previously been isolated from the seeds of *Psoralea corylifolia* [20]. Chen et al., studies [21] on *P. corylifolia* extracts have reported that corylin plays a role in SIRT activation, contributing to lifespan extension and regulation of lipid metabolism in mice. Additionally, corylin is known for its antioxidant, anti-aging, and antitumor properties. Its therapeutic potential has been demonstrated in various conditions, including hyperlipidemia, insulin resistance, atherosclerosis, hepatocellular carcinoma, and neurological disorders. Notably, this study is the first to highlight the skin-whitening effects of corylin isolated from APPL.

Melanin synthesis is stimulated by α-MSH, which binds to melanocortin receptor 1 (MC1R), activating adenylate cyclase and increasing intracellular cyclic AMP (cAMP) levels. Elevated cAMP promotes the expression of tyrosinase, TRP1, and TRP2 and induces the transcription of MITF, a key transcription factor involved in melanin production and transport. Tyrosinase catalyzes the initial and rate-limiting steps of melanin biosynthesis, converting tyrosine to dopaquinone. TRP1 and TRP2, two key enzymes in the later stages of melanogenesis, play distinct roles: TRP1 primarily facilitates the oxidation of DHICA (5,6-dihydroxyindole-2-carboxylic acid) to its corresponding quinone, contributing to eumelanin formation, whereas TRP2 functions as a dopachrome tautomerase, converting dopachrome into DHICA to regulate the balance between eumelanin and pheomelanin production. [22]. In this study, corylin significantly inhibited melanin content and intracellular tyrosinase activity in a dose-dependent manner. Additionally, corylin downregulated the expression of TRP1 but had no significant effect on TRP2 expression. This suggests that corylin’s inhibitory effect on melanin synthesis may be attributed, at least in part, to its modulation of TRP1, thereby disrupting eumelanin formation. Since TRP1 is known to stabilize and enhance the activity of tyrosinase, the downregulation of TRP1 by corylin could further contribute to the observed reduction in melanin synthesis. These results highlight the specificity of corylin in targeting tyrosinase and TRP1, reinforcing its potential as a melanogenesis inhibitor.

To further elucidate the molecular mechanism, we examined the effect of corylin on the expression of genes and proteins associated with melanin production.

Various molecular mechanisms regulate melanin synthesis [22]. Activation of the MEK/ERK pathway impacts melanin production by phosphorylating MITF, thereby affecting its activity and stability. Similarly, the Akt/GSK-3β signaling pathway downregulates MITF expression, reducing the transcription of tyrosinase and TRP-1 Various molecular mechanisms regulate melanin synthesis. Activation of the MEK/ERK pathway impacts melanin production by phosphorylating MITF, thereby affecting its activity and stability [23]. Similarly, the Akt/GSK-3β signaling pathway downregulates MITF expression, reducing the transcription of tyrosinase and TRP-1 [24]. Previous studies have shown that compounds such as Methyl Gallate [25]and 4-Methylcoumarin [26] decrease melanin synthesis by modulating the ERK and Akt pathways.

Arbutin, known for its well-characterized ability to inhibit melanin production, was used as a positive control [27]. It suppresses melanin synthesis by competitively binding to the active site of tyrosinase, thereby blocking the early stages of melanin biosynthesis. As a widely recognized reference compound for evaluating skin-whitening agents, arbutin served as a benchmark for comparing the inhibitory effects of corylin on melanin synthesis and tyrosinase activity.

Tyrosinase undergoes activation through glycan modification mediated by α-glucosidase, transitioning into its active form. α-Glucosidase inhibitors interfere with the proper folding of tyrosinase into its active conformation, leading to its delivery to melanosomes in an inactive, copper-deficient state, thereby suppressing melanin synthesis [28]. Additionally, corylin has been reported to inhibit the glucose trimming process required for tyrosinase activation, effectively blocking the glycosylation of tyrosinase. Corylin demonstrated superior inhibitory activity against α-glucosidase compared to the positive control, acarbose. [29]. These findings suggest that corylin represents a novel mechanism for melanin synthesis inhibition via α-glucosidase suppression, highlighting its potential as an effective skin-whitening agent.

Traditional approaches to cosmetic skin efficacy studies have relied on a progression from in vitro cell-based assays to animal testing and, ultimately, human trials. However, growing ethical concerns about the suffering of animals used in cosmetic testing have led to widespread regulatory changes. Since the European Union's ban on animal testing for cosmetics in 2008, most countries have implemented similar prohibitions, prompting the development of alternative testing models.

Artificial skin models, constructed using tissue engineering techniques, are composed of living human skin cells and structural components like collagen. These models closely mimic the structure and biological responses of human skin, offering a reliable and ethically sound alternative to animal testing. As a result, artificial skin models have gained recognition as an effective bridge between in vitro cell studies and clinical trials due to their high relevance to human physiology [30, 31].

An artificial skin model comprising an epidermal layer with melanocytes was utilized in this study. UVB irradiation was applied to induce melanin synthesis, serving as an alternative to the conventional α-MSH treatment. UV radiation is known to promote mitochondrial elongation and increase melanin production. UVB-stimulated artificial skin models replicate the histological changes observed in human skin following UV exposure, including sunburn cell formation, erythema, and changes in epidermal thickness. These models also exhibit modulated cytokine profiles linked to pigmentation, apoptosis, DNA repair, and aging mechanisms.

Notably, research by Visalini Muthusamy [32] has highlighted the role of the p38 MAPK pathway in regulating TNFα release in UV-irradiated melanocyte-derived cells, underscoring the complex signaling involved in UV-induced responses.

UVB-irradiated artificial skin models were employed to investigate the effects of corylin on melanin synthesis. Assessments of melanin content and Fontana-Masson (F-M) staining demonstrated that corylin also reduced melanin synthesis in UVB-stimulated reconstructed human 3D skin models. This approach not only provides a robust platform for studying pigmentation-related pathways but also offers insights into the potential therapeutic applications of corylin in managing hyperpigmentation and photoaging.

## Conclusion

Corylin was shown to exhibit significant potential as a novel skin-whitening agent by effectively inhibiting melanin synthesis through multiple mechanisms. It suppressed tyrosinase activation by targeting α-glucosidase and modulated key signaling pathways, including MEK/ERK and Akt/GSK-3β, to downregulate melanin-related gene expression. Its efficacy was further validated in UVB-stimulated 3D reconstructed skin models, reinforcing its application in mitigating hyperpigmentation and photoaging. These findings underscore corylin's promise as an innovative alternative to conventional whitening agents. Further investigations are warranted to confirm its clinical safety and therapeutic efficacy in dermatological applications.

## Experimental

### Materials

Dulbecco’s Modified Eagle Medium (DMEM), penicillin and streptomycin (PC/SM), phosphate-buffered saline (PBS), and fetal bovine serum (FBS) were obtained from Life Technologies Corporation. Acarbose, α-glucosidase (from *S.cerevisiae*), p-nitrophenyl-a-D-glucopyranoside (pNPG), α-melanocyte-stimulating hormone (α-MSH), dimethyl sulfoxide (DMSO), L-DOPA, 3-(4,5-dimethylthiazol-2-yl)-2,5-diphenyltetrazolium bromide (MTT), LY294002, PD98059, and SB216763 were purchased from Sigma-Aldrich (St. Louis, MO, USA). The Easy-Blue™ Total RNA extraction kit was purchased from Intron Biotechnology, Inc. (iNtRON Biotechnology, Seongnam, Korea). The TaqMan® RNA-to-Ct™ 1-Step Kit was purchased from Applied Biosystems (USA). RIPA buffer was obtained from Thermo Fisher Scientific (Waltham, MA, USA). Protease inhibitor cocktail tablets were purchased from Roche Diagnostics (Switzerland). Antibodies were purchased from Cell Signaling Technology (Danvers, MA, USA) and Santa Cruz Biotechnology Inc. (Santa Cruz, CA, USA). The ECL Western Blotting Analysis System was obtained from Amersham Biosciences (Piscataway, NJ, USA).

### Extraction and isolation

The dried and pulverized aerial part of *P. lobata* (10 kg) were extracted under reflux with 30% ethanol at 90 °C for 3 h. The solvent was removed under reduced pressure to give a residue (2.02 kg), which was suspended in distilled water (DW) and partitioned with ethyl acetate (EtOAc) to obtain the EtOAc extract of the aerial part of *P. lobata* (APPL, 145 g). APPL, activated carbon and EtOAc were suspended at a ratio of 1:1.5:7, filtered, and then the solvent was removed under reduced pressure to obtain a decolored extract (GALM-DC, 58 g). Then, GALM-DC was suspended in DW and partitioned with CHCl_3_ to give the CHCl_3_ extract (10.1 g). The CHCl_3_ extract was subjected to column chromatography (CC) on silica gel eluting with a gradient of CHCl_3_-MeOH (18:1 to 0:1, v/v) to give fourteen fractions (Fr.1-Fr.14). Fr.C3 (1.79 g) was performed on a sephadex LH-20 CC eluted with CHCl_3_ to afford eleven subfractions (Fr.C3.1-C3.11). Fr.C3.11 (1.1 g) was firstly performed on a silica gel CC eluted with hexane–EtOAc (4:1, v/v) to get three sub-fractions (Fr.C3.11.1-Fr.C3.11.3). Then, Corylin (18.5 mg) was yielded from Fr.C3.11.3 (46.8 mg) by recrystallization and decolorization with methanol. Compounds isolated from Fr.C3.11.3 were identified as corylin from 1H- and 13C-NMR spectral data (Palo Alto, CA, USA).

Corylin—Light yellow powder; ^1^H-NMR (DMSO-*d*_6_, 400 MHz) δ: 10.79 (1H, s, 7-OH), 8.33 (1H, s, H-2), 7.98 (1H, d, *J* = 8.8 Hz, H-5), 6.95 (1H, dd, *J* = 8.8, 2.4 Hz, H-6), 6.87 (1H, d, *J* = 2.4 Hz, H-8), 6.80 (1H, d, *J* = 8.0 Hz, H-5’), 7.28 (1H, d, *J* = 2.0 Hz, H-2’), 7.31 (1H, dd, *J* = 8.0, 2.4 Hz, H-6’), 5.78 (1H, d, *J* = 9.6 Hz, H-3’’), 6.44 (1H, d, *J* = 10.0 Hz, H-4’’), 1.40 (6H, s, 2’’-2CH_3_); ^13^C-NMR (DMSO-*d*_6_,100 MHz) δ: 153.7 (C-2), 123.7 (C-3), 175.1 (C-4), 127.8 (C-5), 115.8 (C-6), 163.2 (C-7), 102.7 (C-8), 158.0 (C-9), 117.2 (C-10), 125.0 (C-1’), 127.5 (C-2’), 121.1 (C-3’), 152.8 (C-4’), 116.1 (C-5’), 130.2 (C-6’), 76.8 (C-2’’), 131.8 (C-3’’), 122.3 (C-4’’), 28.3 (C-5’’), 28.3 (C-6’’).

### Cell culture and cell viability assay

B16F10 murine melanoma cells were obtained from ATCC (VA, USA), and cultured in DMEM supplemented with 10% FBS and 1% antibiotics in 5% CO_2_ at 37℃.

B16F10 cells (1 × 10^4^ cells/well) were seeded into a 96-well plate and incubated for 24 h. Cells were treated with fraction and corylin for 48 h. The medium was suctioned and 1 mg/mL MTT solution was added and incubated for 2 h. After that, formazan was dissolved in DMSO and absorbance was read at 540 nm using a microplate reader (BioTek, VT, USA).

### Melanin content determination

B16F10 cells (1 × 10^5^ cells/well) were seeded in a 6-well plate and exposed to α-MSH (100 nM) for 24 h. Cells were treated with a combination of α-MSH, fraction and corylin for 48 h. Cells were washed twice with PBS and lysed in 400 μL of 1N NaOH for 1 h at 90℃. The resulting melanin concentration was quantified by measuring the absorbance at 405 nm. Melanin production was expressed as a percentage of that from α-MSH-treated controls.

### Measurement of cellular tyrosinase activity

B16F10 cells were exposed to α-MSH (100 nM) for 24 h and treated with a combination of α-MSH and corylin for 48 h. The pellet was obtained by washing with PBS. The cells were lysed with 0.1 M sodium phosphate buffer (pH 6.8) containing 5 mM EDTA, 1% Triton X-100, and 0.1% phenylmethylsulfonyl fluoride (PMSF) in ice for 30 min. After centrifugation of the lysate at 13,000 rpm for 30 min at 4 °C, cellular tyrosinase activity was measured in the resulting supernatant. Enzyme activity was normalized to protein concentration, as determined by the Bradford assay. The cellular tyrosinase and 0.1% L-DOPA reaction was performed in 0.1 M sodium phosphate buffer at 37 °C for 1 h. Tyrosinase activity was quantified by measuring the absorbance at 475 nm.

### Western blot analysis

B16F10 cells were treated with corylin and then lysed with RIPA buffer to obtain a protein. 30 μg of proteins were electrophoresed by 7.5% SDS‑PAGE gels and transferred to nitrocellulose.

The membranes were blocked with 5% skim milk in PBST (PBS containing 0.1% Tween 20) at RT for 1 h, washed with PBST, incubated with primary antibodies (1:1,000) for 18 h at 4˚C, washed with PBST, incubated with HRP-conjugated secondary antibodies for 2 h at RT. The protein-antibody complex was visualized by an ECL system. Bands were quantified using the FluorChem E system image analyzer (Cell Biosciences, CA). β-actin was used as an internal control.

### Measurement of MITF, TRP-1, and tyrosinase mRNA expression

The mRNA was quantified by ND-1000 spectrophotometer (NanoDrop Technology, USA). The relative ratio of target gene expression was calculated with the △Ct method. The transcripts of selected genes were quantified by RT-PCR using a TaqMan RNA-to-Ct 1-Step Kit according to the manufacturer’s instructions. Reverse transcription was performed at 48 °C for 15 min followed by the activation of AmpliTaq Gold DNA Polymerase at 95 °C for 10 min, and 40 cycles of amplification at 95 °C for 15 s and 60 °C for 1 min on an ABI Step One Plus (Applied Biosystem, USA).

### α-Glucosidase inhibition assays

The α-glucosidase enzyme inhibition assay was performed according to the method described by Si et al. [33]. Briefly, 10 μL of test samples at various concentrations (31.3- 250 μM), 20 μL of α-glucosidase (0.5 unit/mL), and 120 μL of 0.1 M phosphate buffer (pH 6.9) were mixed. After incubating at 37℃ for 15 min, 20 μL of pNPG (5 mM) was put into substrates, which were incubated for an additional 15 min. The reaction was terminated by the addition of 80 μL of 200 mM sodium carbonate (Na_2_CO_3_). The absorbance was measured at 405 nm. Acarbose was used as a positive control. The enzyme inhibitory rate was calculated as follows:

Activity (%) = Sample absorption/Control absorption × 100

### Culture of reconstructed 3D skin model

A 3D human skin model (Neoderm-ME) was purchased from Tegoscience Co. (Seoul, Korea). In brief, Neoderm-ME was removed from the medium-containing agar and transferred onto 12-well plates for equilibration at 37 °C in 5% CO_2_ for one day. Treatment of vehicle or sample (corylin or arbutin), for 1 h before UVB (50 mJ/cm) irradiation for eight days. The medium was changed every day and the plate was incubated at 37 °C and 5% CO_2_. Then, the skin tissues were used to determine melanin content and subjected to Fontana-Masson (F-M) staining.

#### Fontana-masson staining

To observe the degree of skin pigmentation, F-M staining was performed. 3D human skin tissue blocks were fixed with 4% formalin for 12 h and embedded in paraffin. Sections cut to 5 µm thickness were stained using the F-M staining kit from IHC World (Woodstock, MD, USA).

F-M staining was performed according to the manufacturer’s protocol. The stained tissues were observed with a Leica phase contrast microscope (Leica Microsystems, Wetzlar, Germany) to measure the epidermal thickness. The thickness of the epidermis was evaluated using the average of three measurements from each tissue.

### Statistical analysis

Statistical calculations were performed with SigmaPlot software, version 10.0 (Systat Software Inc., San Jose, CA, USA). The results are expressed as the mean ± SD. Student's *t*-test was used for statistical analyses; *p*-values < 0.05 were considered statistically significant.

## Data Availability

The data supporting the results of this study can be obtained from the corresponding authors upon reasonable request.
